# Zinc and Iron Homeostasis: Target-Based Drug Screening as New Route for Antifungal Drug Development

**DOI:** 10.3389/fcimb.2019.00181

**Published:** 2019-05-29

**Authors:** Claudia Simm, Robin C. May

**Affiliations:** ^1^School of Biosciences, Institute of Microbiology and Infection, University of Birmingham, Birmingham, United Kingdom; ^2^Department of Biochemistry and Molecular Biology, Monash University, Clayton, VIC, Australia

**Keywords:** *Candida*, antifungals, high throughput drug screening, zinc homeostasis, iron homeostasis, artemisinin, pyrvinium pamoate

## Abstract

The incidence of fungal diseases is on the rise and the number of fatalities is still unacceptably high. While advances into antifungal drug development have been made there remains an urgent need to develop novel antifungal agents targeting as-yet unexploited pathways, such as metal ion homeostasis. Here we report such an approach by developing a metal sensor screen in the opportunistic human fungal pathogen *Candida albicans*. Using this reporter strain, we screened a library of 1,200 compounds and discovered several active compounds not previously described as chemical entities with antifungal properties. Two of these, artemisinin and pyrvinium pamoate, have been further characterized and their interference with metal homeostasis and potential as novel antifungal compounds validated. Lastly, we demonstrate that the same strain can be used to report on intracellular conditions within host phagocytes, paving the way toward the development of novel screening platforms that could identify compounds with the potential to perturb ion homeostasis of the pathogen specifically within host cells.

## Introduction

The incidence of life-threatening fungal infections is increasing dramatically, primarily due to the rise in patients with impaired immunity through conditions like HIV/AIDS, primary immune deficiency, cancer chemotherapy or organ transplantation (Roemer and Krysan, 2014). Most current antifungal drugs were developed in the 1980s and target either ergosterol or cell wall biosynthesis. Limited efficacy and substantial host toxicity mean that systemic fungal disease is amongst the deadliest human infection, exhibiting mortality rates in excess of 50%. Together, the leading fungal pathogens in humans (*Candida albicans, Aspergillus fumigatus*, and *Cryptococcus neoformans*) cause over two million infections, and a million deaths, per year (Pianalto and Alspaugh, 2016). In addition, increasing resistance toward these “old drugs,” as well as the emergence of intrinsically resistant fungal pathogens such as *Candida auris* means that the development of new therapeutic strategies for this group of organisms is paramount (Chowdhary et al., 2017).

The understanding of fungal pathogenesis has increased, and new virulence factors have been identified over the recent years (Perfect, 1996; Nicola et al., 2018). So far none of these fungal virulence genes have been exploited as a druggable target in *Cryptococcus* spp. and *Candida* spp. (Wong et al., 2014).

Lately, much attention has been paid to gene products involved in ion homeostasis (Li et al., 2018). Several metal-regulated or metal-regulatory genes such as *PRA1, FTR1, CSR1*, and *HAP43* have been identified as virulence factors and loss of function of these genes attenuates virulence in animal models (Staats et al., 2013; Ding et al., 2014; Gerwien et al., 2017; Skrahina et al., 2017).

Inside host phagocytic cells, pathogenic organisms face extreme iron and zinc limitation. These essential metal ions are actively withheld by the host by tightly regulating the level of labile ions to very low concentrations, thereby limiting their availability to microbial pathogens (Potrykus et al., 2014). The host protects metal ions by compartmentalization or through the activity of metal-binding proteins such as hemoglobin, transferrin, calprotectin, and lactoferrin (Philpott, 2006; Gerwien et al., 2017). Invasive fungi have to scavenge these ions in order to secure sufficient levels of iron and zinc to proliferate under limiting conditions. Microorganisms use an array of high- and low-affinity metal transporters and their expression is regulated by metal-responsive transcription factors. Those include the *C. albicans* zinc-responsive transcription factor Csr1/Zap1 and the iron-responsive counterparts Sef1 and Hap43 (Kim et al., 2008; Noble, 2013).

Zinc is a critical micronutrient for fungal growth and the regulation of its uptake and sequestration has been well studied in the model yeast *S. cerevisiae*. This budding yeast has both high (i.e., Zrt1) and low (i.e., Zrt2) affinity zinc transporters which are regulated by the Zap1 transcription factor. Zap1 recognizes zinc-responsive elements in the promoter regions of zinc-regulated genes (Eide, 2003). In metal replete conditions, this transcription factor inhibits expression of Zap1-regulated genes while transcription is triggered under zinc deficiency conditions. Homologous zinc transporters as well as their transcriptional regulators are present in a wide range of pathogenic fungi (Jung, 2015). *C. albicans* also contains two zinc uptake transporters Zrt1 and Zrt2. Unlike *S. cerevisiae*, in *C. albicans* these zinc uptake proteins cannot be described as low and high affinity transporters, but their expression is pH-dependent (Crawford et al., 2018). While Zrt1 facilitates uptake under neutral to alkaline conditions, Zrt2 is the main uptake transporter in *Candida* over a wider pH range. It is essential for infection and a *ZRT2* deletion mutant showed reduced virulence in a murine candidiasis model. Transcription level of both *ZRTs* are regulated by the transcription factor Csr1 (Kim et al., 2008; Böttcher et al., 2015). A *C. albicans csr1/csr1* mutant showed severe defects in growth and filamentation and Csr1 is described as an important virulence factor.

Most fungal organisms rely on two iron uptake mechanisms: (1) a membrane-bound reductase/permease complex and (2) internalization of extracellular Fe^3+^-siderophore complexes (Philpott, 2006). Siderophores are small high-affinity iron chelators. The fungal pathogens *Cryptococcus* spp. and *Candida* spp. cannot synthesize their own siderophores but rather encode siderophore–iron transporters and can utilize siderophores synthesized by bacteria or other fungi (Heymann et al., 2002). The reductive iron uptake pathway is best characterized in *S. cerevisiae*, where iron uptake is facilitated through a Fet3/Ftr1 complex (Kosman, 2003). Fet3 is a multi-copper ferroxidase which reduces ferric iron to ferrous iron which is taken up into the cell by the ferrous permease Ftr1. The deletion of the *FTR1* homologs in *C. albicans* (*CaFTR1*) and *C. neoformans* (*CFT1*) lead to impaired iron acquisition and attenuated virulence in a mouse model (Ramanan and Wang, 2000; Jung et al., 2009). Both high affinity iron transport pathways are strictly regulated depending on metal availability in the surrounding environment. Sef1 and Hap43 are main iron uptake regulators under iron limiting conditions (Chen et al., 2011; Skrahina et al., 2017).

In this study we sought to identify drugs that disrupt essential micronutrient, zinc and iron homeostasis in fungal pathogens by interfering with metal uptake, transcriptional regulation or sequestration processes within pathogens such as *C. albicans*. We designed fluorescence-based zinc and iron sensors by fusing zinc and iron responsive promoter elements to GFP or dTomato reporters. We show that the resulting metal sensors are amenable to high throughput drug screening and respond rapidly to changes in micronutrient limitation. We used *C. albicans* harboring the metal sensors to screen 1,200 compounds of the Prestwick Chemical library for novel antifungals. The total hit rate did not exceed 2.5%, indicative of a target-specific high throughput platform. Hit compounds were validated via secondary assays for a metal-dependent mode of action, whilst a phagocyte-based assay demonstrated the feasibility of using these promoter-reporter sensors for the identification of drugs with increased anti-microbial activity in phagocytes.

Taken together, this study presents a method for the identification and verification of new antifungal drugs targeting the perturbation of zinc and iron homeostasis using *C. albicans* as a model fungal pathogen.

## Methods

### Strains and Media

The *C. albicans CaI4* strain *ura3*::imm434/*ura3*::imm434 *iro1/iro1*::imm434 was used in this study and grown in YPD at 30°C. Yeast transformants carrying the zinc or iron sensor were plated on YNB plates without uridine. Positive transformants were grown in liquid YNB-ura and all high throughput screens and validation experiments were carried out in phenol red-free and serum-free RPMI-1640 (R8755, Sigma). The medium was buffered with 3.5% MOPS and the pH was adjusted to either pH 5.6 for growth at 30°C (yeast) or to pH 7.2 for growth at 37°C (hyphae). The cloning of the metal sensors was carried out *in Escherichia coli* strain *DH5*α (New England Biolabs) which was grown in LB with or without ampicillin.

### Generation of the Zinc and Iron Promoter-Reporter

Standard cloning procedures were used for the DNA manipulation and transformation of *E. coli*. The genes of GFP and dTomato were amplified from plasmid template pGFP-RH (gift from Rebecca Hall, Hall et al., 2011) and genomic DNA from a dTomato expressing *Candida* strain (gift from Rebecca Hall), respectively. The promoter sequences of *ZRT2* and *HAP43* were amplified from genomic DNA from *CaI4*. All primers are shown in Table 1. PCR products were digested with relevant restriction enzymes (Table 1) and inserted into *pGFP-RH*. The resulting plasmids were linearized with the restriction enzyme XhoI before integration into the *URA3* site of the *CaI4* genome using the lithium acetate protocol. Transformants were plated out onto YNB-ura and grown for 2–3 days before testing for GFP and dTomato expression.

**Table 1:** Primer sequences and restriction sites.

**Primer**	**Sequence**	**Restriction site**
CaZrt2prom 5′	CGCG GCGGCCGC ATATTGTGTAATTTTACATTT	NotI
CaZrt2prom 3′	CGCG ACTAGT TCAGTGTTAACTAATTC	SpeI
Hap43prom 5′	CGCG GCGGCCGC AGAATGTTACTAGATTCT	NotI
Hap43prom 3′	CGCG ACTAGT GTTCAAATTGAAATTC	SpeI
dTomato 5′	CGCG ACTAGT ATGGTTTCAAAAGGTG	SpeI
dTomato 3′	CGCG GGATCC TTATTTATACAATTCATCC	BamHI
GFP 5′	CGCG ACTAGT ATGTCTAAAGGTGAAG	SpeI
GFP 3′	CGCG GGATCC CTAGCTTATTTGTACAA	BamHI

### Validation of Assay Conditions

Transformants carrying the zinc or iron sensor were inoculated into RPMI medium (pH 5.6 or 7.2) and grown with or without metal chelators DTPA, EDTA, and TPEN (Sigma) for up to 24 h at 30 or 37°C, respectively. Cells were adjusted to a starting cell density OD_600_ of 0.05 and suspensions were transferred into black, clear bottom 96-well plates (Corning CLS3603, Sigma). Cell density absorbance at 600 nm, GFP (485-12 ex/520 em) and dTomato (544 ex/620-10 em) expression were measured every 2 h using a FLUOStar Omega (BMG labtech) plate reader.

### High Throughput Screen and Hit Compound Validation

The Prestwick Chemical library containing 1200 FDA-approved compounds was used for this screen. The 10 mM compounds stocks were diluted in 40% DMSO to a concentration of 400 μM in intermediate plates. Five microliters of the intermediate plate solutions were spotted into black clear bottom 96-well assay plates (Corning CLS3603, Sigma) using a Microlab Star Liquid Handling System (Hamilton). Immediately 200 μl of *C. albicans* suspension adjusted to a cell density OD_600_ = 0.05 in RPMI, pH 7.2 was added to the assay plates. A negative DMSO and a positive 5 μM TPEN control was added to each assay plate. For the high throughput screen *Candida* expressing the Zrt2promoter-dTomato and Hap43promoter-dTomato were used. The cell density (OD_600_) and dTomato (544ex/590 em) were measured after 14 h of incubation at 37°C without shaking using a POLARStar Omega, (BMG labtech).

Identified hit compounds were purchased from Sigma and serially diluted as indicated. Cultures of *C. albicans* transformed with Zrt2promoter-dTomato, Hap43promoter-dTomato, Zrt2promoter-GFP, and Hap43promoter-GFP were diluted to a cell density OD_600_ = 0.05 in RPMI, pH 5.6 or 7.2 and incubated at 30 or 37°C, respectively. Cell density, GFP, and dTomato fluorescence were measured between 12 and 14 h.

### Time-Lapse Experiments

*Candida* cell expressing the zinc or iron promoter reporter were grown in black, clear bottom 96-well plates (Corning CLS3603, Sigma) at room temperature in RPMI, pH 5.6 for 24 h with or without drug treatment, and GFP and dTomato fluorescence was monitored using the JuLI Stage Real-Time Cell History Recorder (NanoEnTek, Korea). Images were taken every hour for 24 h using the transmitted light, GFP (466-40 ex/525-50 em) and RFP (525-50 ex/580LP em) channel at a 20-fold magnification.

### Toxicity Assay

The lung epithelial cell line A549 was grown in RPMI-1640 (21875, Gibco) with 10% FBS and seeded at 4,000 cells per well in a 96-well plate. After 1 day of incubation at 37°C, 5% CO_2_ cultures were treated with a serial dilution of selected drugs and incubated for a further 48 h. Fifteen microliter of CellTiterBlue (Promega) was added to each well including medium only controls and incubated for 2–4 h and fluorescence (544 ex/580 em) was immediately measured using a plate reader (FLUOStar Omega, BMG labtech).

### Measurement of Total Metal Content

*C. albicans* cell cultures were grown in YNB for 16 h with or without drug treatment. Cells were harvested and washed in ice-cold 1 mM EDTA. The cell pellet was then dried in a vacuum concentrator (Concentrator 5301, Eppendorff) and its dry weight was measured. The dry cell pellet was digested in 200 μl 65% nitric acid (Suprapur, Merck) and 15 μl H_2_O_2_ at 70°C for 30 min. The digest was filled up to a total volume of 5 ml with distilled water and subjected to inductively coupled plasma—optical emission spectrometry ICP-OES measurement using an ICP-OES spectrometer (OES Optima 8000 and S10 Autosampler, Perkin Elmer).

### Measurement of Labile Zinc

*C. albicans* cell cultures were grown in RPMI, pH 5.6 at 30°C for 16 h with or without drug treatment. The zinc dye FluoZin-3AM (ThermoFisher) was added to the samples at a final concentration of 5 μM and incubated for a further 30 min at 30°C. Cells were washed twice in PBS and fluorescence was immediately measured using a flow cytometer (NxT Attune, ThermoFisher). The FluoZin-3AM fluorescence was measured using the FITC channel.

### *Candida* Phagocytosis Assay

J774 macrophages grown in low glucose DMEM (D5546, Sigma) with 10% FBS were plated at cell density of 1 × 10^5^ cells per well onto sterile 18 mm glass microscopy cover slips (VWR) in a 24-well plate and incubated at 37°C with 5% CO_2_ overnight. Macrophages were activated by adding 1 ml of serum-free DMEM medium containing 15 μl of a 10 μg/ml phorbol 12-myristate 13-acetate (PMA) stock (P8139, Sigma). Macrophages were incubated for 1 h at 37°C and 5% CO_2_. Immediately medium was removed and cells were washed twice in PBS. Overnight cultures of *C. albicans* containing the fluorescent metal sensors were washed in PBS and adjusted to a cell density of either 2 × 10^6^ or 5 × 10^6^ cells/ml. Nine hundred and fifty microliter of fresh serum-free DMEM with 50 μl of the *Candida* suspension was added to the macrophages and incubated for a further hour at 37°C and 5% CO_2_. After the incubation the medium was removed, cells were washed twice with PBS followed by fixation in 4% paraformaldehyde for 10 min. The fixative was removed, cells were washed twice in PBS and mounted onto microscopy slides with Fluoromount (F4680, Sigma). Images were taken at 40 × magnification using a Zeiss Axio observer microscope using DIC, GFP, and Texas Red channel. Images were acquired through Axio Zen software (Zeiss).

## Results

### Metal Chelators Increase GFP Fluorescence in a Time-Dependent Manner

**Figure 1:**
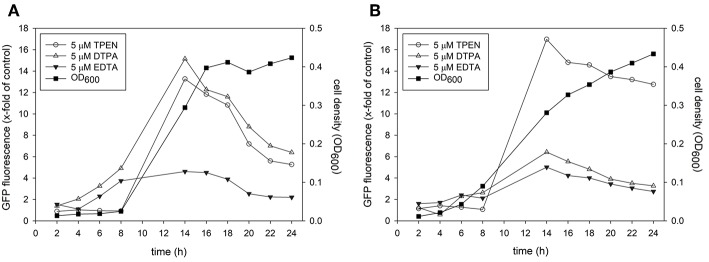
A culture of *Candida albicans* containing the *Zrt2prom*-GFP promoter-reporter construct was adjusted to a cell density OD_600_ = 0.05 and grown in either **(A)** RPMI, pH 5.6 at 30°C or **(B)** RPMI, pH 7.2 at 37°C. Cells were grown in absence or presence of indicated metal chelator and cell density and GFP expression was measured every 2 h.

For the development of metal responsive reporter-promoter constructs, the promoter sequences of the zinc-regulated *ZRT2* and the iron-regulated *HAP43* of *C. albicans* were fused to GFP or dTomato. In previous studies the expression of these genes was up-regulated under zinc or iron starvation and essential for metal uptake under metal-limiting conditions (Jung, 2015; Skrahina et al., 2017; Crawford et al., 2018). We therefore expected an increase in fluorescence expression when labile intracellular concentration of zinc and iron decreased. The zinc and iron promoter-reporter constructs were integrated into the genome of C*. albicans* to avoid copy number effects associated with episomal expression. Since the identification of hit compounds relies on the ability of *Candida* sensor strains to detect metal limitation, we grew them in the presence of metal chelators (Figure 1). The expression of GFP in *Candida* containing the Zrt2prom-GFP sensor was significantly increased 12 h after transfer into chelator containing medium. The chelators DTPA and TPEN strongly induced GFP under yeast growth conditions (RPMI, pH 5.6 and 30°C) whereas EDTA showed only weak induction (Figure 1A). TPEN caused the biggest increase in fluorescence under hyphal growth conditions (RPMI, pH 7.2 and 37°C) while both DTPA and EDTA showed a much weaker GFP expression (Figure 1B). The highest GFP expression was observed between 12 and 14 h, in close agreement with previous studies in which *Saccharomyces cerevisiae* as well as *C. albicans* could sustain proliferation for eight cell division cycles (e.g., 12–14 h) without external zinc (Simm et al., 2007; Duncan Wilson, personal communication). This time frame was also associated with the highest z'-factors and therefore used as the end point read-out for the high-throughput assay. At later time points the signal window declines, something that is particularly prominent under yeast growth condition. At these later time points, cells deplete their internal zinc storage and therefore experience zinc starvation, associated with an induction of GFP expression which in turn effects the dynamic range of the signal window. A sharper decline of the signal window is seen in yeast cultures which reach stationary phase earlier than hyphae.

**Figure 2:**
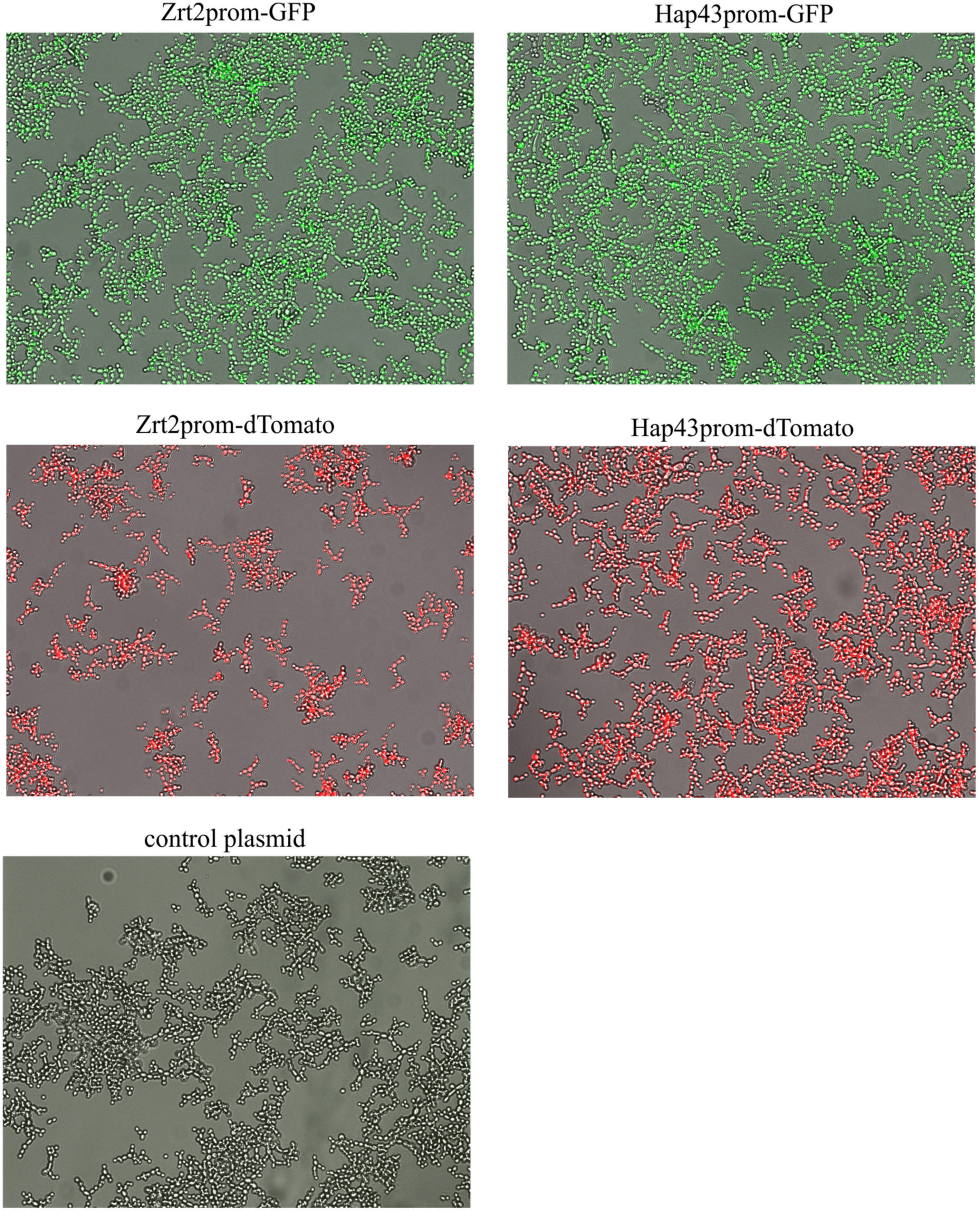
*Candida albicans* containing the metal promoter-reporter construct or a control plasmid were adjusted to a cell density OD_600_ = 0.05 and grown in RPMI, pH 5.6 at room temperature with 5 μM TPEN for 24 h. Images were taken by JuLI Stage Real-Time Cell History Recorder (NanoEnTek, Korea) every hour. Images shown were taken at 16 h after treatment.

In order to test if all promoter-reporter constructs generated for this study are induced similarly we observed growth and fluorescence expression in a Real-Time Cell History Recorder (NanoEnTek, Korea) for 24 h. Due to the lack of a temperature-controlled incubation chamber in the Cell History Recorder cells had to be grown at room temperature. Fluorescence was initially triggered at 6 h and fully expressed by 12 h for all transformed strains tested (Figure 2, Supplementary Movies). No fluorescence was seen for *Candida* transformed with a control plasmid (Figure 2). Additionally, fluorescence in untreated vs. treated condition was investigated. Only a very small population of untreated yeast expressed dTomato (Supplementary Movie 3) or GFP (data not shown) after 6 h of growth. In contrast to the metal chelator treated samples only a marginal increase in fluorescence intensity was observed thereafter. Metal chelator treated samples steadily increased fluorescence, suggesting that the GFP and dTomato signal is a response to lower level in iron and zinc availability and thus that these strains represent specific sensors for a metal-dependent high throughput screen.

### Screening of the Prestwick Chemical Library

We screened the established Prestwick Chemical library which consists of 1200 FDA-approved small compounds to test the potential of the zinc and iron sensor to identify novel antifungal compounds. The original idea of a double sensor strain containing the green fluorescent zinc sensor (Zrt2prom-GFP) and the red fluorescent iron sensor (Hap43prom-dTomato) had to be abandoned. Due to the brightness of the dTomato fluorescence its signal bled into the GFP channel in plate reader experiments. Therefore, the zinc and iron sensor had to be analyzed separately. For the high throughput screen the Zrt2prom-dTomato and Hap43prom-dTomato promoter-reporter strains were used since the z'-factors were higher than those of Zrt2prom-GFP and Hap43prom-GFP strains. *Candida* strains were grown at 37°C for 12–14 h, since these conditions resulted in the best z'-factor (>0.5). Moreover, a temperature of 37°C has greater biological relevance than growth at 30°C. All compounds were tested at a final concentration of 10 μM in 1% DMSO. Every assay plate contained a negative control (1% DMSO) which set the baseline of dTomato expression and a 5 μM TPEN positive control to assess quality control and plate-to-plate variability.

**Figure 3:**
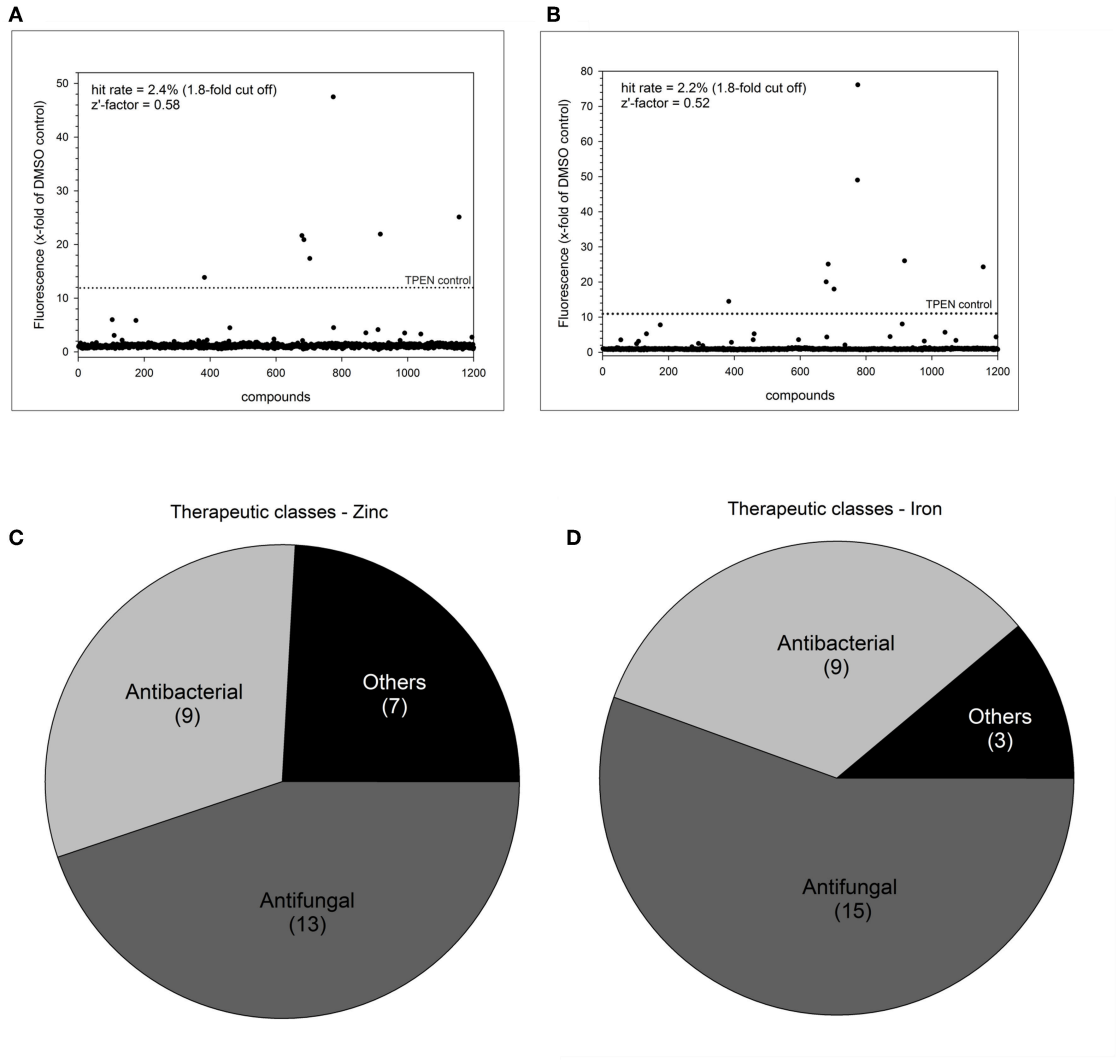
Summary of high throughput metal sensor screen. The total hit distribution and therapeutic classes for the zinc sensor **(A,C)** and iron sensor **(B,D)** are shown.

We selected a cut-off value of 1.8-fold for expression of dTomato compared to the DMSO baseline level, resulting in an overall low hit rate of 29 compounds (2.4%) for zinc and 27 compounds (2.2%) for iron (Figures 3A,B). The number of drug hits is much reduced compared to the *S. cerevisiae* metal-sensor screen which was conducted at a compound concentration of 100 μM (Simm et al., 2011), suggesting that this higher concentration may have triggered significant off-target effects. The z'-factor over all 15 plates for the zinc and iron screen was 0.58 and 0.52, respectively and therefore met the criteria for an excellent assay with a large separation of positive and negative signal readouts (Iversen et al., 2006). We grouped the hits from this screen according to their therapeutic classes, with most compounds being classified as antibacterial or antifungal (Figures 3C,D). These compounds were of less interest to us due to their previous use in antimicrobial therapy. We instead turned our focus on the compounds with no previous association with antimicrobial properties. Additionally, two small molecules (Merbromin and Doxorubicin) which showed a very high dTomato signal in the high throughput screen were excluded as false-positives due to their red color and high autofluorescence. We further restricted the hit compound list by excluding directly fungicidal compounds that resulted in a viability of < 10%, since metal-dependent regulatory processes are strongly disturbed in dying cells. A full summary of all hit compounds can be found in Supplementary Table 1. All compounds that were exclusively defined as hits for only zinc or only iron are listed in Table 2.

**Table 2:** HTS compounds unique for zinc or iron.

**Zinc**	**Iron**
Nifurtimox	Voriconazole
Tenatoprazole	Miconazole
Clofazimine	Quinacrine HCl
Pemirolast potassium	Propidium iodide
Artemisinin	

### Validation of Selected Hit Compounds

**Figure 4:**
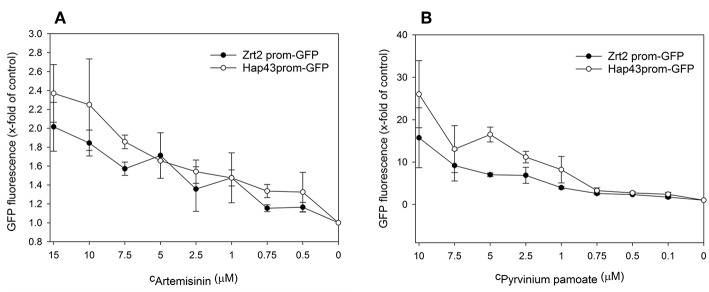
A culture of the promoter-reporter strains of *Candida albicans* were adjusted to a cell density OD_600_ = 0.05 and grown in RPMI, pH 7.2 at 37°C for 16 h with indicated concentrations of **(A)** Artemisinin or **(B)** Pyrvinium pamoate when cell density (OD_600_) and GFP fluorescence was measured. The values are the means ± standard deviation (SD) of at least 3 independent experiments.

**Figure 5:**
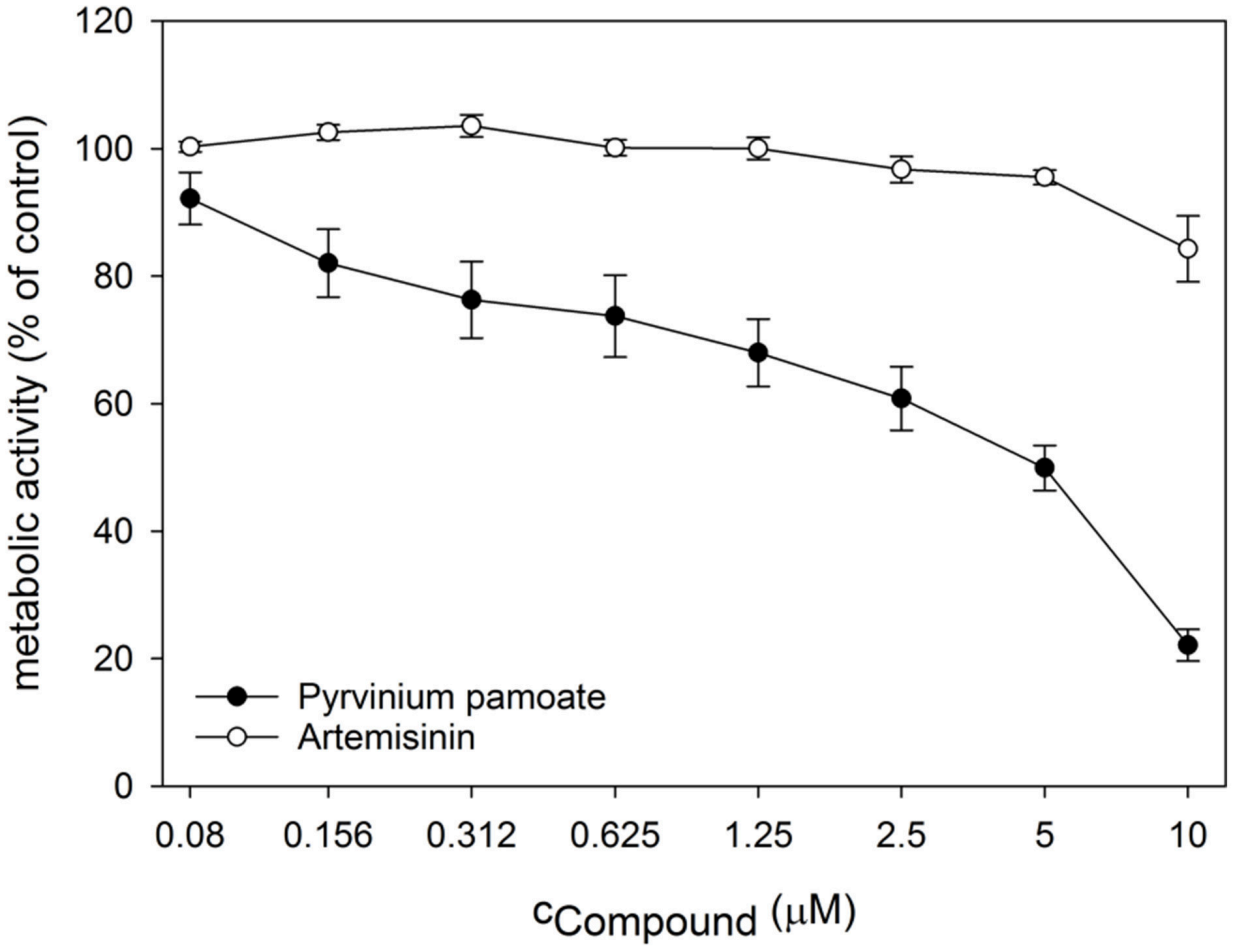
Cell cultures of A549 epithelial cells were treated with indicated concentrations of Artemisinin and Pyrvinium pamoate for 48 h. Metabolic activity was determined by CellTiterBlue assay. The values are the means ± SD of at least 3 independent experiments.

Next, we performed dose response analyses to validate lead compounds that have not previously been reported to have antimicrobial activity. Tenatoprazole did not confirm as a hit and Nifurtimox only triggered increased fluorescence expression at concentrations of 0.25–1 μM which is far below the initial screening concentration. Both compounds were not followed up further. The hits artemisinin, an antimalarial, as well as pyrvinium pamoate, an anthelmintic, were selected for further validation studies. Pyrvinium pamoate showed autofluorescence due to its inherent red color. However, its intrinsic fluorescence was smaller than the dTomato fluorescence readout. In order to avoid any interference of background fluorescence all dose response experiments were carried out with the GFP metal sensors where pyrvinium pamoate did not show any autofluorescence. Figure 4 shows the dose response curves for both compounds, artemisinin and pyrvinium pamoate, with a significant increase in GFP expression from 7.5 μM for artemisinin and 1 μM for pyrvinium pamoate. We then tested the toxicity of these compounds in a human cell line. The lung epithelial cell line A549 was grown with increasing concentrations of artemisinin and pyrvinium pamoate for 48 h (Figure 5). While only a slight reduction in metabolic activity could be observed at 10 μM Artemisinin, pyrvinium pamoate started to have growth limiting effects at much lower concentration with an IC_50_ of 5 μM.

### Artemisinin and Pyrvinium Pamoate Effect Intracellular Zinc and Iron Level

**Figure 6:**
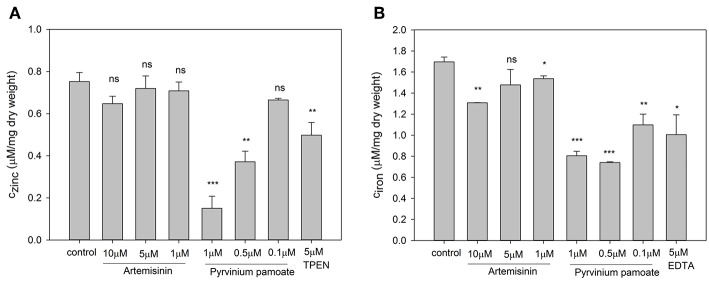
*Candida albicans* cultures were adjusted to a cell density OD_600_ = 0.01 and grown for 16 h in YNB with indicated compound concentrations and total cellular **(A)** zinc and **(B)** iron content was measure via ICP-OES. The values are the means ± SD of at least 2 independent experiments with 2 technical replicates. Changes of total metal content was compared to control conditions and *p*-values were calculate by unpaired, two tailed *t*-test (ns *p* > 0.05, ^*^p ≤ 0.01, ^**^p ≤ 0.001, ^***^p < 0.001).

In order to test the specificity of the metal sensors we measured the intracellular metal content upon drug treatment. The total zinc and iron concentration were determined by ICP-OES. For this experiment cells had to be grown in YNB rather than RPMI, since the very low basal metal concentration in the latter medium is too close to the detection limit of the ICP-OES instrument. Artemisinin has no effect on total intracellular zinc (Figure 6A) but caused a small but significant reduction in intracellular iron (Figure 6B) at 1 μM and a further significant decrease at 10 μM when compared to control conditions. Pyrvinium pamoate reduces the total intracellular zinc concentration to a level below seen for the zinc chelator TPEN at concentrations of 0.5 and 1 μM (Figure 6A). This effect is lost when reducing the concentration to 0.1 μM. It also significantly reduces intracellular iron level at all concentrations tested (Figure 6B). Again, drug concentrations of 0.5 and 1 μM pyrvinium pamoate drop metal concentrations below those measured for the metal chelator EDTA.

**Figure 7:**
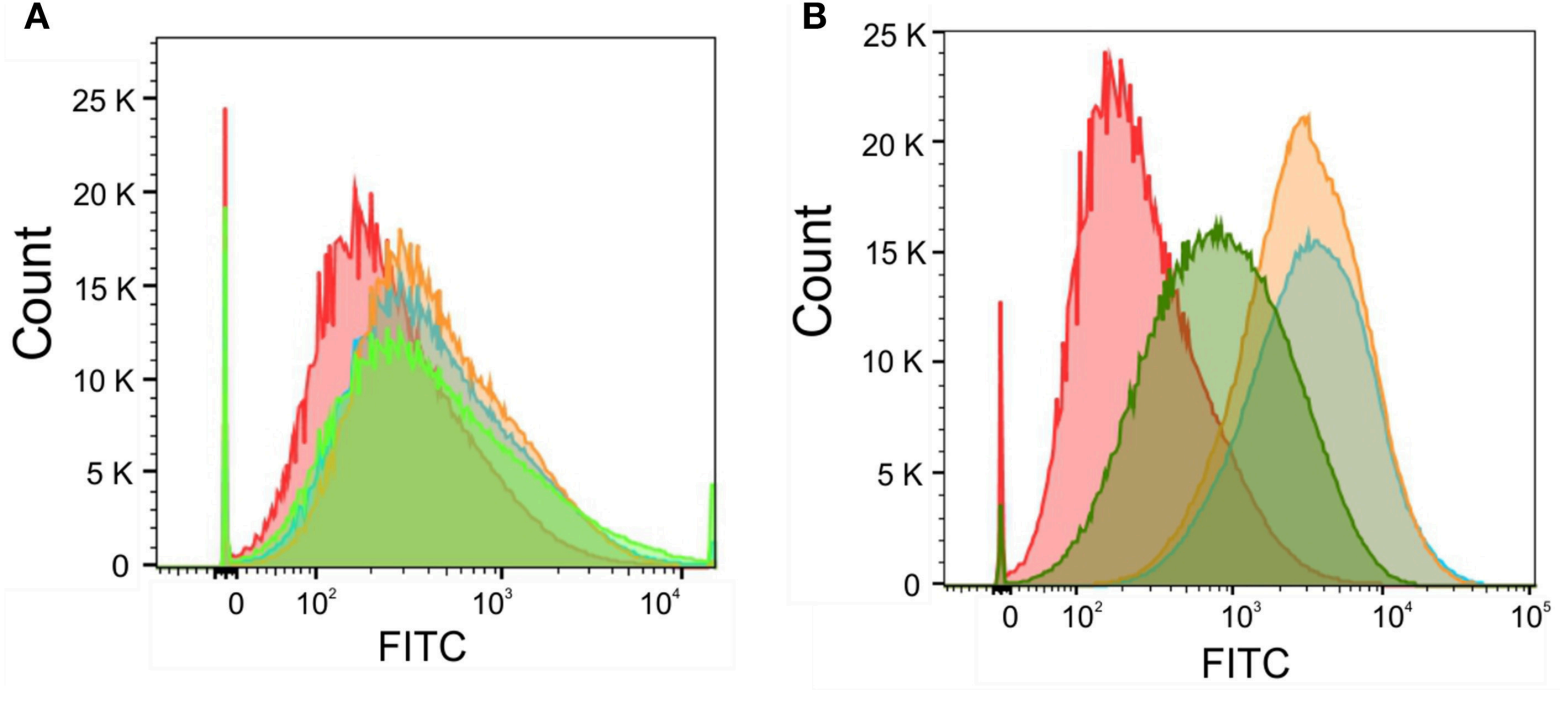
*Candida albicans* were adjusted to a cell density OD_600_ = 0.05 and grown in RPMI, pH 5.6 at 30°C for 16 h with **(A)** Artemisinin (10μM—orange, 5μM—blue, 1 μM—green, or DMSO—red) or **(B)** Pyrvinium pamoate (1μM—orange, 0.5μM—blue, 0.1μM—green, or DMSO—red). Cells were stained with 5 μM FluoZin-3AM and labile zinc concentration was measured via flow cytometry.

Next, we wanted to correlate the obtained total intracellular zinc and iron concentrations with labile intracellular zinc and iron level by using metal fluorescent indicators. By staining *C. albicans* wildtype with the zinc dye Fluo-Zin3AM a small but significant shift in fluorescence intensity could be observed for all artemisinin-treated samples, suggesting that labile zinc concentrations were decreased (Figure 7A). An increase in fluorescence intensity of nearly two orders of magnitude could be detected in samples treated with 1 and 0.5 μM pyrvinium pamoate (Figure 7B), which correlates strongly with the reduction in total cellular zinc seen for these concentrations. Unfortunately, the iron probe experiment was unsuccessful and therefore only data for labile zinc using can be shown at this point.

### Phagocytosis of *Candida* Metal Sensor Strains

**Figure 8:**
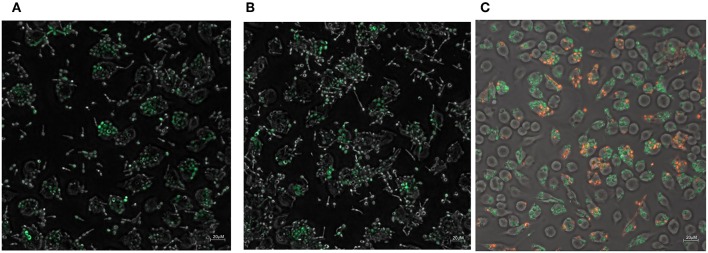
The phagocytosis of *Candida albicans* transformed with **(A)** the Zrt2prom-GFP, **(B)** Hap43prom-GFP or **(C)** 1:1 mixture of *Candida albicans* transformed with either Hap43prom-dTomato or Zrt2prom-GFP was monitored at 1 h after exposure to J774 cells and images were obtained with a Zeiss Axio microscope using a 40 × objective.

The zinc and iron promoter-reporter strains can provide a powerful tool for many applications and thus we tested their application in a phagocytosis assays using J774 macrophages. *Candida* carrying Zrt2prom-GFP or Hap43prom-dTomato were engulfed by macrophages within 1 h and showed good expression of the fluorescent reporter within these host cells (Figures 8A,B). Due to the lack of a double zinc/iron promotor-reporter strain, a 1:1 mixture of *C. albicans* transformed with either Hap43prom-dTomato or Zrt2prom-GFP was used to monitor changes in fluorescence for both sensors in one macrophage cell (Figure 8C). *Candida* carrying either metal sensor were detected within one macrophage without any interferences seen in the plate reader experiments.

The addition of artemisinin or pyrvinium pamoate to the inoculum did not alter GFP or dTomato expression (data not shown). However, the drug treatment could only be applied for 1 h (since *Candida* filaments and destroys the host cell after that time point) a time frame that might be too short for the impact of the drug to be revealed. Preliminary data suggest that the metal sensor fluorescence in *Candida* within the macrophages is higher than in those not phagocytosed (Figures 8A,B). Again, a longer incubation of the pathogen with macrophages is needed to fully evaluate this finding. The introduction of a mutant partially impaired in hyphae formation (Δ*cph1*) did not sufficiently extent the assay time and hyphae formation was observed at 4 h after the inoculation of *Candida* to macrophages (data not shown).

## Discussion

Currently, only three distinct antifungal drug classes—azoles, polyenes, and echinocandins—are in current clinical use. This is greatly surpassed by the number of drugs available for viral and bacterial diseases. While improvements in antifungal drug development have been made, none of the current antifungal compounds act on fungal-specific targets such as virulence factors. The most common virulence factors are involved in processes such as adhesion, morphogenesis, phenotypic switching, biofilm formation, and metabolic adaptation (Mayer et al., 2013; Gerwien et al., 2017). The latter includes trace metal acquisition which has been described as a promising pathway for novel antifungal drug development (Simm et al., 2011; Bernardo et al., 2014; Li et al., 2018). Exploiting pathogen specific virulence factors would exert less selective pressure on pathogenic fungi, thereby minimizing effects on the host system and reducing the selective pressure for drug resistance compared to growth inhibiting drugs.

We developed a high throughput screen to identify previously unknown antifungal drugs. Unlike the classical approach, this screen will not identify compounds with fungicidal properties, but is designed to target zinc and iron homeostasis in the opportunistic fungal pathogen *C. albicans*. Drugs identified in this way will increase fungal susceptibility to host nutritional immunity and can strengthen existing monotherapies with known antifungal agents.

The screening of the Prestwick Chemical library with 1200 FDA approved small molecules revealed 29 and 27 potential hits perturbing zinc or iron homeostasis, respectively (Figure 3). Most of them were already classified as antibacterial or antifungal drugs, e.g., azoles (Supplementary Table 1). Azole antifungals can bind metals due to their chemical structure and have been shown to interfere with metal homeostasis (Seward et al., 2006; Silva et al., 2011). Five compounds had not previously been investigated for antifungal therapy and were pursued further.

After secondary validation, two compounds (artemisinin and pyrvinium pamoate) emerged as lead compounds. Artemisinin is primarily described as an antimalarial drug but recently its pharmacological properties have been extended to antitumor, antibacterial, antiviral, antileishmanial, antischistosomiatic, and herbicidal activities (Krishna et al., 2008; Liu et al., 2019). While its exact mode of action is not yet fully understood, several studies investigated ferrous iron binding as a plausible mechanism for this drug (O'Neill et al., 2010; Shandilya et al., 2013). In line with this, we observed a reduction in total intracellular iron as well as labile zinc with this drug (Figures 6B, 7A). Reduction in the labile zinc pool was previously observed in *A. fumigatus, Fusarium*, and *C. albicans* treated with antimalarial drugs such as atovaquone and halofantrine (Simm et al., 2011; Clark et al., 2018). However, no reduction in total zinc accumulation could be detected in *Candida* under these conditions. These findings are similar to the result in this study where the total zinc accumulation did not change upon treatment with artemisinin. This may seem contradictory regarding the increase in fluorescence seen for the Zrt2prom-GFP and Zrt2prom-dTomato promoter-reporter constructs. The zinc sensor is sensitive to the available or labile zinc concentration that can be used in biological processes, rather than the total zinc concentration. Since a reduction in the labile zinc pool could be measured, we believe that artemisinin changes the availability of labile zinc while total zinc concentrations remains unchanged. The drug might interfere with compartmentalization or remobilization of the intracellular zinc pool or affect zinc chelation. In those events the intracellular labile zinc would not be accessible, and the cell would sense zinc deficiency which in turn would trigger transcription of zinc responsive genes such as *ZRT2*.

The second lead compound, pyrvinium pamoate, is an anthelmintic effective for the treatment of pinworms but repurposing strategies have revealed its potential in cancer therapy (Momtazi-borojeni et al., 2018). Pyrvinium pamoate did show an adverse effect on metabolic activity in A549 cells at concentrations higher than 2.5 μM (Figure 5), however, it has been approved by FDA for the safe use in humans and is used as a therapeutic with few side effects. The drug is thought to interfere with glucose uptake but also contributes to the inhibition of mitochondrial respiration complex 1 (Sheth, 1975; Ishii et al., 2012). Recent advances in cancer therapy revealed that pyrvinium pamoate suppresses the Wnt signaling cascade (Li et al., 2014). No reference to metal binding or interfering with metal regulatory processes could be found but our data strongly suggest its involvement in zinc and iron homeostasis (Figures 6A,B, 7B).

Both artemisinin and pyrvinium pamoate show fungistatic rather than fungicidal properties [Supplementary Table 1, (Siles et al., 2013)]. For this reason, both compounds will only be effective in adjunct (ideally synergistic) therapy with known antifungals such as azoles. Synergistic drug combination is a promising alternative approach to discover drugs with unexploited chemical scaffolds and a unique mechanism of actions. Synergism can potentially reduce the dose of single drug usage with increased drug efficacy, and subsequently lower the drug toxicity and development of drug resistance. De Creemer et al. conducted a high throughput screen for miconazole potentiators against *C. albicans* biofilm formation and identified both artemisinin derivatives and pyrvinium pamoate as candidate molecules (De Cremer et al., 2015). Artemisinins in particular exhibited highly synergistic efficacy when combined with miconazole. Similarly, a synergistic effect was found in combination with itraconazole against *A. fumigatus* (Gautam et al., 2011). The black yeast *Exophiala dermatitidis* was treated with a combination of pyrvinium pamoate and itraconazole, posaconazole, or voriconazole and synergistic effects could be observed for all three combinations (Gao et al., 2018). Several studies on adjunct treatment of metal chelators, such as EDTA, dibromoquinoline, or DIBI, and azoles showed markedly reduced susceptibility of fungi toward these combinations in comparison with antifungal monotherapy (Casalinuovo et al., 2017; Mohammad et al., 2018; Savage et al., 2018). While most of these studies focus on iron metabolism, targeting zinc homeostasis has also been proven to be a successful strategy (Cohrt et al., 2018).

Consequently, targeting zinc and iron metabolism opens up new opportunities for novel therapeutic strategies. The discovery of small molecules that interfere with iron and zinc homeostasis will expand the chemical diversity of antifungals and will lead to enhanced efficacy of currently administered antifungal drugs. The zinc and iron sensors established in this work have successfully identified new chemical entities with antifungal properties. They provide a strong tool for further antifungal screens and can be easily adapted to other pathogenic fungi such as *Cryptococcus* spp. or *Aspergillus* spp. Our results invite further studies on these promoter-reporter constructs to explore metal-dependent activation within phagocytes. Compounds that increase depletion of iron or zinc in pathogenic fungi inside macrophages or neutrophils could facilitate increased killing and clearance.

## Data Availability

The datasets generated for this study are available on request to the corresponding author.

## Author Contributions

CS established link to industry partner and funding. CS designed and conducted the experiments, analyzed the data, and wrote and revised the publication. RM supervised and provided additional funding.

### Conflict of Interest Statement

The authors declare that the research was conducted in the absence of any commercial or financial relationships that could be construed as a potential conflict of interest.

## Supplementary Material

Supplementary Table 1 Summary of HTS hit compounds.

Supplementary Movie 1 Time-lapse experiment of *Candida albicans* transformed with Zrt2prom-GFP treated with 5 μM TPEN.

Supplementary Movie 2 Time-lapse experiment of *Candida albicans* transformed with Hap43prom-dTomato treated with 5 μM TPEN.

Supplementary Movie 3 Time-lapse experiment of *Candida albicans* transformed with Hap43prom-dTomato without treatment.
