# Supplementary material for: Zinc and Iron Homeostasis: Target-Based Drug Screening as New Route for Antifungal Drug Development
Source: Front Cell Infect Microbiol. 2019 May 29;9:181. doi: 10.3389/fcimb.2019.00181 (PMC6548825; doi:10.3389/fcimb.2019.00181)
Supplement: Supplementary Table 1 — Summary of HTS hit compounds. [file Table_1.DOCX]

**Supplementary table 1.** Summary of HTS hit compounds

| **Chemical name** | **x-fold induction** | | **growth** | **Therapeutic effect** |
| --- | --- | --- | --- | --- |
|  | **zinc** | **iron** | **(% of control)** |  |
| Antimycin A | 5.82 | 7.77 | 67 | Antibacterial, Antifungal |
| Artemisinin | 1.82 | 0.96 | 76 | Antimalarial |
| Butoconazole nitrate | 1.98 | 3.53 | 35 | Antibacterial, Antifungal |
| Chlorhexidine | 5.98 | 2.40 | 13 | Antibacterial, Antiseptic |
| Clofazimine | 2.04 | 1.17 | 86 | Antibacterial |
| Clotrimazole | 1.93 | 2.50 | 34 | Antibacterial, Antifungal |
| Doxorubicin hydrochloride | 4.46 | 5.23 | 84 | Antibacterial, Antineoplastic, Immunosuppressant |
| Enilconazole | 2.12 | 3.15 | 43 | Antifungal |
| Fluconazole | 2.72 | 4.34 | 34 | Antifungal |
| Flucytosine | 4.13 | 8.02 | 18 | Antifungal |
| Isoconazole | 2.16 | 5.23 | 30 | Antifungal |
| Ketoconazole | 2.20 | 2.80 | 34 | Antifungal |
| Miconazole | 1.72 | 3.51 | 31 | Antifungal |
| Nifurtimox | 3.53 | 0.85 | 34 | Antiprotozoal |
| Oxiconazole Nitrate | 2.08 | 4.31 | 33 | Antifungal |
| Propidium iodide | 1.28 | 2.05 | 92 | Antibacterial |
| Pyrvinium pamoate | 3.30 | 5.67 | 35 | Anthelmintic |
| Quinacrine dihydrochloride hydrate | 1.14 | 1.88 | 59 | Anthelmintic, Antileishmanial, Antimalarial, Antiparasitic, Antiprotozoal |
| Sertaconazole nitrate | 1.80 | 3.36 | 37 | Antibacterial, Antifungal |
| Sulconazole nitrate | 3.53 | 4.44 | 41 | Antifungal |
| Tenatoprazole | 2.38 | 1.29 | 54 | Antiulcer |
| Tioconazole | 3.04 | 3.10 | 31 | Antifungal |
| Voriconazole | 1.71 | 3.57 | 31 | Antifungal |
